# Forecasting the Incidence of Mumps in Zibo City Based on a SARIMA Model

**DOI:** 10.3390/ijerph14080925

**Published:** 2017-08-17

**Authors:** Qinqin Xu, Runzi Li, Yafei Liu, Cheng Luo, Aiqiang Xu, Fuzhong Xue, Qing Xu, Xiujun Li

**Affiliations:** 1 Department of Biostatistics, School of Public Health, Shandong University, Jinan 250012, China; xu_qinqin@126.com (Q.X.); runzi_li@hotmail.com (R.L.); liuyafei@mail.sdu.edu.cn (Y.L.); luomadcheng@hotmail.com (C.L.); xuefzh@sdu.edu.cn (F.X.); 2 Shandong Center for Disease Control and Prevention, Jinan 250014, China; aqxuepi@163.com

**Keywords:** mumps, time series analysis, SARIMA model, infectious disease epidemiology

## Abstract

This study aimed to predict the incidence of mumps using a seasonal autoregressive integrated moving average (SARIMA) model, and provide theoretical evidence for early warning prevention and control in Zibo City, Shandong Province, China. Monthly mumps data from Zibo City gathered between 2005 and 2013 were used as a training set to construct a SARIMA model, and the monthly mumps in 2014 were defined as a test set for the model. From 2005 to 2014, a total of 8722 cases of mumps were reported in Zibo City; the male-to-female ratio of cases was 1.85:1, the age group of 1–20 years old accounted for 94.05% of all reported cases, and students made up the largest proportion (65.89%). The main serious endemic areas of mumps were located in Huantai County, Linzi District, and Boshan District of Zibo City. There were two epidemic peaks from April to July and from October to January in next year. The fitted model SARIMA (0, 1, 1) (0, 1, 1)_12_ was established (AIC = 157.528), which has high validity and reasonability. The SARIMA model fitted dynamic changes of mumps in Zibo City well. It can be used for short-term forecasting and early warning of mumps.

## 1. Introduction

Mumps is an acute respiratory infectious disease caused by the mumps virus and characterized by the inflammation of the parotid or other salivary gland. The main symptoms are nonsuppurative swollen and painful glands, low-grade fever, and headache [1,2]. Most cases of mumps are mild and self-limited, but some serious complications can also occur when the virus invaded various glandular tissue, such as the nervous system, liver, kidney and heart. Approximately 10% of mumps cases develop complications [3], with orchitis (testicular inflammation) and aseptic meningitis being the most common [1,4]. Incidents of orchitis were reported in 11.8% of male mumps patients in the Czech Republic [3]. Other complications should not also be neglected, such as pancreatitis, myositis, and oophoritis [5]. In addition, mumps usually occurs in school-age children and adolescents, and can result in childhood deafness [6]. It is also reported that the proportion of mumps cases among adults has also increased [7]. Generally, the complications would be more severe with age, and affect significantly more men than women [8]. Though cases occur in every month, mumps have obvious seasonal characteristics. Outbreak peaks from April to June and from October to January, and occurs regularly at intervals of two to five years [2]. A person would develop symptoms after direct contact with mumps patients after about two to three weeks. The disease is generally transmitted through the respiratory tract by direct contact, droplets, and saliva inhalation; the widespread non-immunized population are susceptible [5].

In China, mumps is defined as a notifiable infectious disease. The annual incidence is more than 20/100,000 since 2005; and infection rates reached 30/100,000 in 2011 and 2012 (http://cdc.ncmi.cn/Share/index.jsp). Although some measures aimed at mumps have been applied, including vaccine immunization [1], the epidemic situation remains serious due to gene mutation of the mumps virus and China’s huge and highly mobile population. Outbreaks have been observed in many countries, such as Korea in 2013 [9], the United States in 2006 [10], the Czech Republic [2], the United Kingdom, and Belgium [11]. Mumps is extremely severe disease in the Shandong province, and it has the second highest rate in respiratory infectious diseases and [12]. That is a serious public health concern in Zibo City especially, which had the highest reported incidence of mumps among the 17 cities of Shandong in 2012 [13]. It is necessary to fully understand the regularity of mumps in Zibo City, and then model and forecast the disease to provide the scientific theoretical evidences for its prevention and control. For infectious disease, there are many complex affecting factors. Time series analysis is a method of quantitative analysis without regard to the influence of intricate factors, and it could make a scientific quantitative forecast for the future trends of the disease in accordance with historical data and time variables [14]. The autoregressive integrated moving average (ARIMA) model proposed by Box and Jenkins is a commonly statistical method to forecast time series data. It could circumvent many problems in the traditional regression, such as the difficulty in getting detailed data and grasping the influencing factors of the forecasted objects. The ARIMA model has been increasingly used in epidemiologic research to describe the temporal pattern of many diseases, such as dengue [15], tuberculosis [16,17], malaria [18] and others [19,20,21].

In this study, the demographic characteristics and spatiotemporal distribution of mumps in Zibo City are described. The seasonal ARIMA (SARIMA) model is established to fit the monthly mumps from 2005 to 2013 in Zibo City, and the fitted model was used to forecast the mumps in 2014 to verify the applicability and feasibility.

## 2. Materials and Methods

### 2.1. Study Area and Data Collection

**Figure 1:**
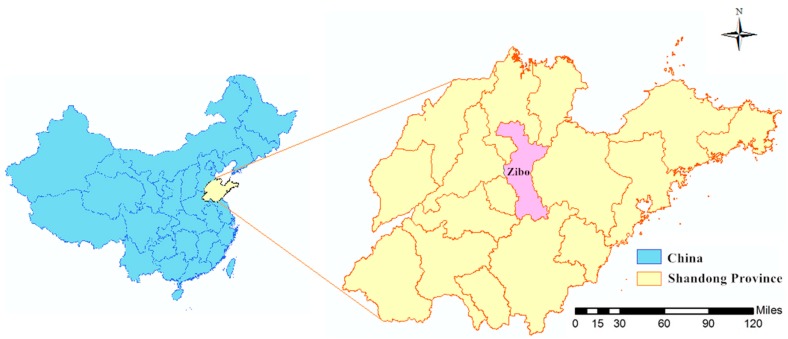
The geolocation of Zibo City in Shandong Province, China (map was created with ArcGIS software, v. 10.2).

Zibo is a central city in the Shandong Province of China, located between latitude 35°55′ N and 37°17′ N, and longitude 117°32′ E and 118°31′ E (Figure 1). The city consists of nine counties with about 4.53 million permanent residents, according to a demographic census in 2010, and a total land area of 5965 square kilometers, which covers nearly 3.8% of Shandong’s entire area. Zibo City has developed into an important modern industrial city with steady population growth.

The data on mumps from 2005 to 2014 in Zibo City are obtained from the Diseases Reporting Information System of the Shandong Center for Disease Control and Prevention, and include the age, sex and occupation for each case. The diagnostic criteria of mumps are the “Diagnostic criteria for mump” established by the Chinese Ministry of Health (http://www.moh.gov.cn/zwgkzt/s9491/200704/38797.shtml), and the disease diagnostic criteria remained consistent during the data collection period.

### 2.2. Statistical Analysis

We used the descriptive epidemiology method to depict the epidemical distribution of mumps firstly, including the temporal and spatial distribution, as well as sex ratio, high-incidence age group, and occupation.

Given that many epidemiologic time series contain significant periodic and seasonal trends, the SARIMA model should be considered, which includes seasonal characteristics of time series [21,22]. The basic structure of a general SARIMA model represents as SARIMA (*p*, *d*, *q*) (*P*, *D*, *Q*)_S_, using the seasonality of mumps as the independent variable and monthly mumps as the dependent variable in the study, and its formula is [21,22]:

$$\nabla^{d}\nabla_{S}^{D}Y_{t}=\frac{\theta_{q}\left(B\right)\Theta_{Q}\left(B^{S}\right)}{\varphi_{p}\left(B\right)\Phi_{P}\left(B^{S}\right)}\varepsilon_{t}$$

$$\varphi_{p}\left(B\right)=1-\varphi_{1}B-\varphi_{2}B^{2}-\ldots \varphi_{p}B^{p}$$

$$\theta_{q}\left(B\right)=1-\theta_{1}B-\theta_{2}B^{2}-\ldots \theta_{q}B^{q}$$

$$\Phi_{P}\left(B^{S}\right)=1-\Phi_{1}B^{S}-\phi_{2}B^{2S}-\ldots \phi_{P}B^{PS}$$

$$\Theta_{Q}\left(B^{S}\right)=1-\Theta_{1}B^{S}-\Theta_{2}B^{2S}-\ldots \Theta_{Q}B^{QS}$$

where *p*, *d* and *q* are the autoregressive order, number of difference, and moving average order, respectively; *P*, *D* and *Q* are the seasonal autoregressive order, number of seasonal difference, and seasonal moving average order, respectively; and *S* is the length of the seasonal period, defined as 12; *B* denotes the backward shift operator, 𝛻=1−B, $Y_{t}\;$ represents the number of mumps at time *t*, and $\varepsilon_{t}$ are the estimated residuals. In the formula, $\varphi_{p}\left(B\right)$ is the *p* order autoregressive coefficient polynomial, $\theta_{q}\left(B\right)$ is the *q* order moving average coefficient polynomial, $\Phi_{P}\left(B^{S}\right)\;$ and $\Theta_{Q}\left(B^{S}\right)$ are the seasonal polynomial functions of order *P* and *Q*, respectively.

The establishment of the SARIMA model is divided into the following four steps [22]: firstly, a time series graph of monthly mumps cases from 2005 to 2014 is drawn to test its stationary intuitively. If the sequence is not smooth, we need to perform logarithmic transformation and differencing on the original sequence. Meanwhile, the Augmented Dickey-Fuller (ADF) method was used to determine whether the new sequence was stable. Secondly, we identified the optional model parameters (*p*, *d*, *q* and *P*, *D*, *Q*) to establish one or more alternative models, according to the autocorrelation coefficient (ACF) and partial autocorrelation coefficient (PACF) of the differenced sequence of the monthly mumps. The conditional least square method was used for parameters estimation. Thirdly, goodness-of-fit tests of models were performed by comparing Akaike’s information criterion (AIC) and Schwarz Bayesian Criterion (SBC). Smaller AIC and SBC indicate the better fitting model [22]. The confirmation of the optimal model must comply with the Ljung-Box *Q* test, which demonstrates that its residual series is a white noise. Finally, the mean absolute percentage error (MAPE) [23], correlation analyses between observed cases and fitted cases, and a fitted and forecasted graph are used to evaluate the accuracy of the final model.

$$MAPE=\frac{1}{n}\overset{n}{\underset{t=1}{\sum }}\left|\left(x_{t}-{\hat{x}}_{t}\right)/x_{t}\right|$$

where $x_{t}$ is the number of reported mumps, ${\hat{x}}_{t}$ is the number of forecasted mumps at time *t*, and *n* presents the number of months for forecasting. In the study, monthly mumps cases from 2005 to 2013 are used as a training set for modeling and fitting a SARIMA model, and monthly data in 2014 are used to forecast the mumps as a test set.

The software SAS 9.4 (Statistical Analysis System, version 9.4, SAS, Cary, NC, USA) was used for statistical analyses in the study, with a two-sided significance level of *p* < 0.05.

## 3. Results

### 3.1. Descriptive Analyses

**Figure 2:**
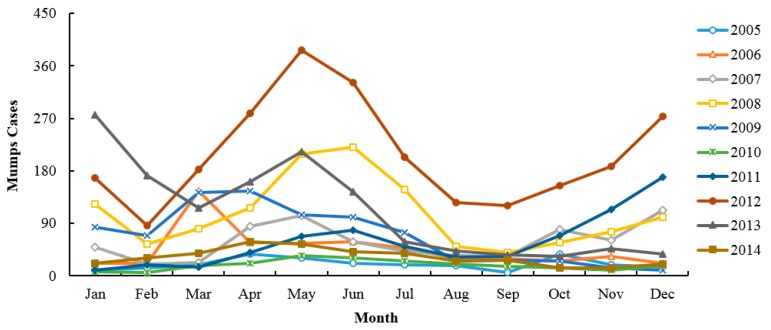
Monthly mumps cases from 2005 to 2014 in Zibo City.

**Figure 3:**
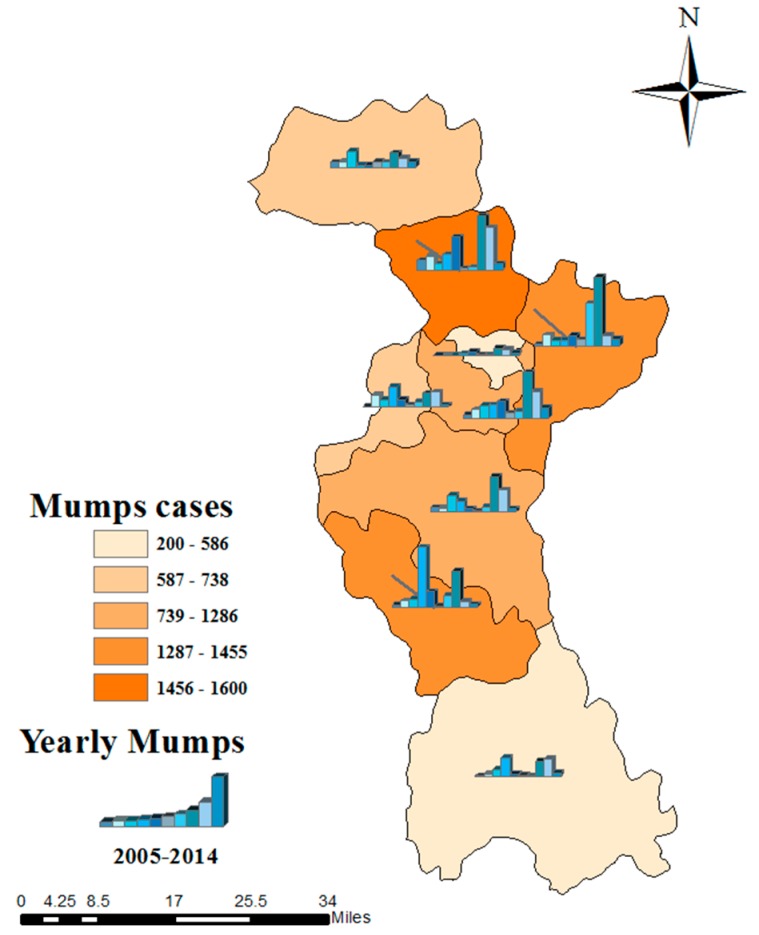
Geographical distribution and yearly mumps cases in Zibo City, from 2005 to 2014.

**Figure 4:**
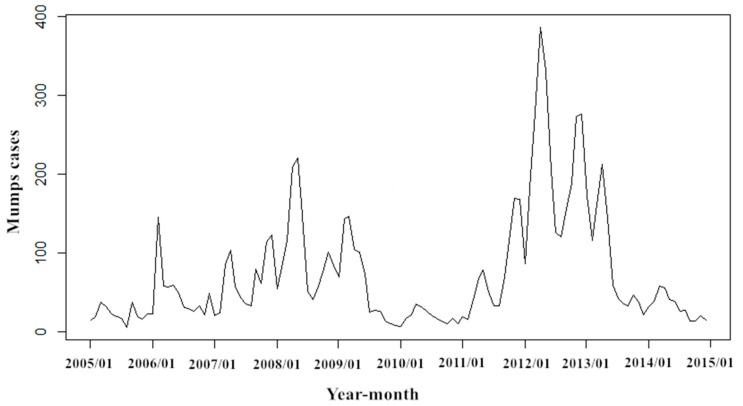
Sequence diagram of monthly mumps cases from January 2005 to December 2014 in Zibo City.

During this study (2005–2014), a total of 8722 mumps cases were reported in Zibo City, Shandong Province, including 5663 males and 3059 females, and a male-to-female ratio of 1.85:1. Mumps mostly occur within the ages of 0–80 years, while the age group of 1–20 accounted for the 94.05% of all reported cases. The highest percentage of mumps cases is found in students, who account for 65.89% (*n* = 5747), followed by the kindergarten children and scattered children. The monthly mumps cases are presented in Figure 2. Though mumps occur in every month, they also have obvious periodicity and seasonality, which manifests basically as two epidemic peaks from April to July and October to January, with an emphasis on the former. In addition, the geographical distribution with annual mumps over a full year and two peak seasons from 2005 to 2014 are shown in Figure 3. Serious endemic areas mainly emerge in Huantai County, Linzi District, and Boshan District. Annual mumps cases are mapped by the histograms; the peak year for most areas in Zibo City is 2012, including Huantai county and Linzi District. Furthermore, there is an outbreak in 2008 in Boshan District. The sequence diagram also show the outbreaks of mumps in 2008, 2012, and 2013 (Figure 4).

### 3.2. SARIMA Model

**Figure 5:**
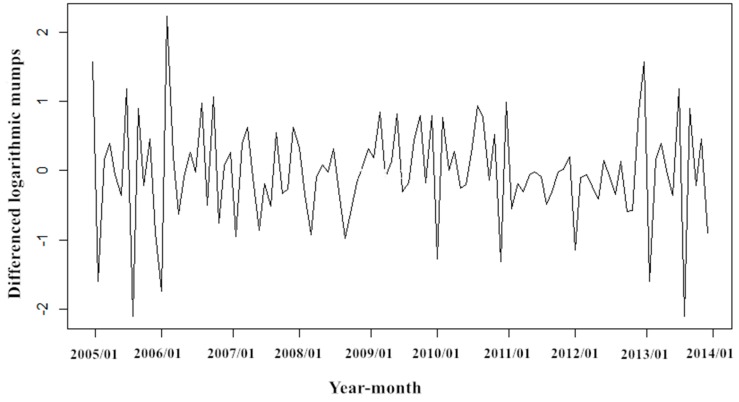
Monthly mumps cases after the logarithmic transformation and difference in Zibo City.

Given the periodicity and seasonality of mumps, we use the natural logarithmic transformation for the fluctuating original sequence to reduce the variance, and then perform a 1-step difference and seasonal difference with a period of 12 to eliminate seasonal trends. The result of the ADF test (*t* = −13.90, *p* < 0.001) indicates that the sequence after difference is stationary, which also is showed in sequence diagram (Figure 5).

**Figure 6:**
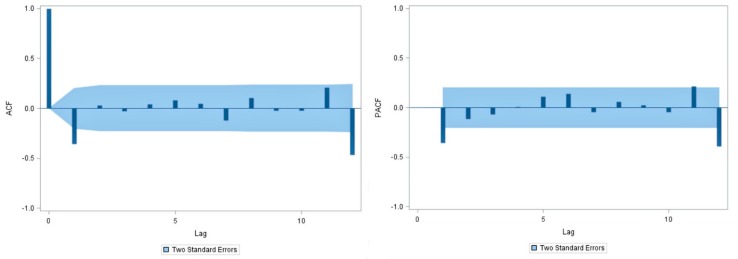
Autocorrelation function (ACF) and partial autocorrelation function (PACF) figures after the logarithmic transformation and difference (the shaded portion is the 95% confidence interval (CI) range).

**Figure 7:**
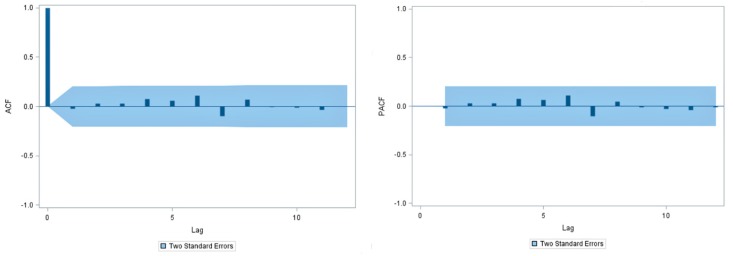
Autocorrelation function (ACF) and partial autocorrelation function (PACF) figures of the residual series (the shaded portion is the 95% CI range).

Figure 6 shows graphs of the ACF and PACF of the transformed and differenced series. Given that autocorrelation coefficients and partial autocorrelation coefficients are both near to zero at all lags that exceed 1, the ACF suggests that *q* should be equal to 0 or 1, and the PACF suggests that *p* is also equal to 0 or 1. Hence, the SARIMA model is considered, and *d* = 1, *D* = 1. Table 1 shows the parameter estimates and their testing results of the SARIMA models which have gotten past parameter verification as well as AIC values, BIC values, and MAPE for models corresponding to different choices of *p*, *q* and *P*, *Q*. According to those results, we hold SARIMA (0, 1, 1) (0, 1, 1)_12_ as the best model, because of the lowest AIC and SBC (483.679, 475.053, respectively). What is more, there is a highest correlation between the observed and fitted forecasted mumps cases on the model (*r* = 0.833, *p* < 0.001). MAPE indicates also that the selected model is feasible. Finally, the results of the Ljung-Box *Q* test (*Q* = 14.85, *p* = 0.869) and residual diagnostic (Figure 7) for the model suggest that the residual series is a ‘white noise’. Therefore, the SARIMA (0, 1, 1) (0, 1, 1)_12_ model could extract fuller information from time series in this study, and is reasonable for forecasting mumps.

**Figure 8:**
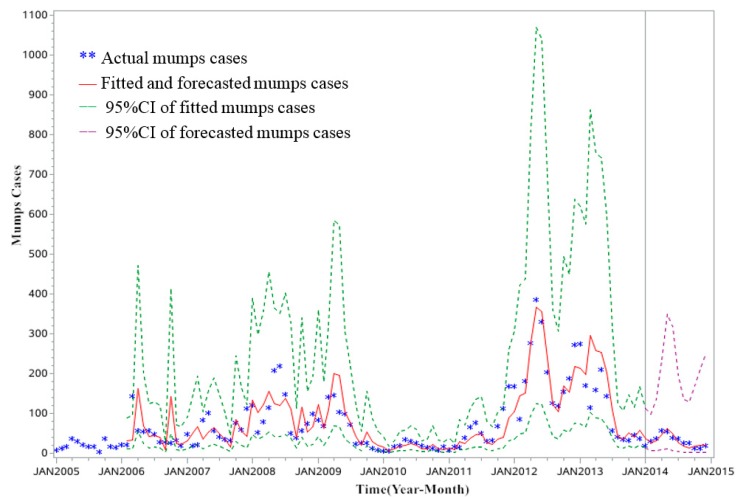
Fitted and forecasted results of monthly cases of mumps in Zibo City (the actual data are contained within the 95% CI of the forecasted value).

Table 2 and Figure 8 shows the comparison of observed and forecasted mumps in Zibo City from January to December in 2014 by the SARIMA (0, 1, 1) (0, 1, 1)_12_ model; the observed cases are all within 95% confidence interval (CI) of the fitted and forecasted values. Furthermore, the forecasted cases are consistent with the changing trend of the observed mumps and match the observed values well, which verified the feasibility and effectiveness of the SARIMA model.

## 4. Discussion

In this study, mumps is one of the most serious infectious disease in Zibo City, and affects nearly two times as many males as females in a total of 8722 reported cases (1.85:1), which is consistent with some previous research results [24,25]. The gender differences in mumps is probably because men were more likely to be exposed to the outside environment than women [25], or because of the higher consultation rate caused by complications occurring more often in males than females [4]. The high peaks regarding age and career of the mumps cases were from 1 to 20 years old (94.05%) and students, respectively, which suggest that the important protection targets ought to be concentrated in children and adolescents. In addition, while the mumps occur year-round, there are two incidence peaks from April to July and from October to January over the next year, showing the obvious seasonal and cyclical characters. During our study, the obvious peak incidence of mumps appears in 2012 and 2013, which may be related to the strengthening of the surveillance and reporting system, the epidemic regularity of mumps itself, and the occurrence of aggregated outbreaks and other related factors [26]. Huantai County and the Linzi and Boshan districts appear to be the high-attack areas in Zibo City, which is in line with another study [25]. This might be related to different demographic characteristics, vaccination coverage, and insufficient measures preventive control measures in schools [25]. The geographic differences contribute to guide the health interventions and allocate health resources reasonably.

Our study finds that the SARMA model (0, 1, 1) (0, 1, 1)_12_ can reflect the incidence regularity of mumps in Zibo City. The model is considered to be reasonable for its short-term forecasting with a high forecasted accuracy based on the MAPE. For the forecasted results, they match the actual data well, but there is little observed difference between observed and forecasted mumps cases in January 2014, which is probably because the main crowds are primary and middle school students. Final examinations in January are bound to result in underestimating the incidence of mumps due to lower rates of visiting the doctor. Hence, this further proves that the SARIMA model is efficient for the disease forecasting. To our knowledge, although there are a few similar studies on the mumps in China [27], this is the first study to apply a SARIMA model for mumps in Zibo City. Our findings demonstrate that the SARIMA (0, 1, 1) (0, 1, 1)_12_ model is an efficient way to forecast the dynamic change of monthly mumps in Zibo City, and it could be used to determine whether the previous epidemic laws have been broken by short-term forecasting. Significantly, early warnings could be provided to health authorities to formulate plans, and implement public health interventions for the prevention and control of the disease.

As one of the extended forms of the ARIMA model, the SARIMA model is particularly suitable for obvious seasonal and periodic surveillance data [28]. In our study, the SARIMA model shows its well-known statistical properties and effective modeling process. Once a satisfactory model is obtained, it can be used to forecast the expected number of cases for any given number of future time intervals. The model can also be easily realized through mainstream statistical software, such as SPSS, SAS, and R [29]. A previous study showed that the best fitted model is the ARIMA model in the various time series methods [30]. Though the exponential smoothing model can also be used to forecast the mumps, the predicted accuracy of the model is generally lower than that of the SARIMA model, and there may be some difficulties in determining the smoothing coefficient. Besides, the exponential smoothing model only predicts at very short intervals, because the weight is progressively smaller with the lengthening of the forecasting term. The SARIMA model demonstrates a stronger forecasting performance than the standard multiple regression model due to the latter’s inability to account for autocorrelations and trends adequately [31]. The artificial neural networks may have the best performance in terms of accuracy, but their specific nonlinear functions within the time series data may not be explained well in practice [29]. Hence, the SARIMA model has a good applying prospect and has been widely used in the forecast of a variety of diseases.

A few limitations about the SARIMA model should be mentioned. Stationarity is the most important requirement for SARIMA model building, namely, the behavior of the time series does not change over time, and varies within a fixed constant mean and variance [14]. Another limitation is that the seasonal parameters estimation should cover at least seven to eight seasonal periods if the sequence contains periodic and seasonal factors; otherwise, the estimated effect will be not ideal [14]. In addition, the SARIMA model applies only to short-term forecasts, as the development of infectious disease is influenced and controlled by many factors. Anyway, we will consider the longer cycles in our future research, such as cross wavelet analysis. Consequently, long-term dynamic observation of mumps is also necessary, which would require updating the time series and re-fit the model or establish a more reasonable model for improving the forecast ability [32]. Furthermore, we will consider the more appropriate models based on various covariates in future research, such as the generalized additive model and distribution lag nonlinear model.

## 5. Conclusions

In summary, the study provides valuable information about epidemic characteristics of mumps in Zibo City. The SARIMA model fits the dynamic changes of mumps well. It is an appropriate statistical model for the prevention and control of the disease by fitting and forecasting surveillance data within a time interval.

## Figures and Tables

**Table 1:** Parameter estimates and testing results, goodness of fits for the seasonal autoregressive integrated moving average (SARIMA) models corresponding to different choices of *p*, *q* and *P*, *Q* which had gotten past parameter verification, as well as correlation analysis between actual and fitted mumps cases.

Models	Parameter Estimation		*t*-test (Parameters Equalization Values With Zero)			Goodness of Fits for Models			Correlation Coefficient	
	Estimate	Standard Error	Lag	*t*	*p*	AIC	SBC	MAPE	*r*	*p*
SARIMA (0, 1, 1) (0, 1, 1)_12_						157.528	165.190	0.297	0.833	<0.001
MA	0.369	0.098	1	3.760	<0.001					
SMA	0.635	0.085	12	7.440	<0.001					
SARIMA (1, 1, 0) (0, 1, 1)_12_						158.413	166.074	0.293	0.831	<0.001
AR	−0.350	0.098	1	−3.550	0.001					
SMA	0.626	0.086	12	7.310	<0.001					
SARIMA (1, 1, 0) (1, 1, 0)_12_						170.668	178.329	0.429	0.813	<0.001
AR	−0.265	0.101	1	−2.620	0.010					
SAR	−0.439	0.097	12	−4.530	<0.001					
SARIMA (0, 1, 1) (1, 1, 0)_12_						170.240	177.901	0.405	0.814	<0.001
MA	0.280	0.101	1	2.770	0.007					
SAR	−0.434	0.097	12	−4.460	<0.001					

Abbreviations: AR: autoregressive; MA: moving average; SAR: seasonal autoregressive; SMA: seasonal moving average.

**Table 2:** Comparison of observed and forecasted mumps from January to December in 2014 by the SARIMA (0, 1, 1) (0, 1, 1)_12_ model.

Time (Month)	January	February	March	April	May	June	July	August	September	October	November	December
Actual Cases	21	31	38	58	55	41	38	26	27	13	13	20
Forecasted Cases	38.63	27.54	32.19	47.82	62.45	49.93	27.41	16.89	14.42	17.00	19.34	20.87
